# Sitosterolemia: Twenty Years of Discovery of the Function of *ABCG5* *ABCG8*

**DOI:** 10.3390/ijms22052641

**Published:** 2021-03-05

**Authors:** Kori Williams, Allison Segard, Gregory A. Graf

**Affiliations:** 1 Department of Pharmaceutical Sciences, College of Pharmacy, University of Kentucky, Lexington, KY 40536, USA; kswilliams3@uky.edu (K.W.); allison.segard@uky.edu (A.S.); 2 Saha Cardiovascular Research Center, Lexington, KY 40536, USA; 3 Barnstable Brown Diabetes and Obesity Center, Lexington, KY 40536, USA

**Keywords:** phytosterol, xenosterol, cholesterol, atherosclerosis, gall stone, ABC, transporter

## Abstract

Sitosterolemia is a lipid disorder characterized by the accumulation of dietary xenosterols in plasma and tissues caused by mutations in either *ABCG5* or *ABCG8*. *ABCG5 ABCG8* encodes a pair of ABC half transporters that form a heterodimer (G5G8), which then traffics to the surface of hepatocytes and enterocytes and promotes the secretion of cholesterol and xenosterols into the bile and the intestinal lumen. We review the literature from the initial description of the disease, the discovery of its genetic basis, current therapy, and what has been learned from animal, cellular, and molecular investigations of the transporter in the twenty years since its discovery. The genomic era has revealed that there are far more carriers of loss of function mutations and likely pathogenic variants of *ABCG5 ABCG8* than previously thought. The impact of these variants on G5G8 structure and activity are largely unknown. We propose a classification system for *ABCG5 ABCG8* mutants based on previously published systems for diseases caused by defects in ABC transporters. This system establishes a framework for the comprehensive analysis of disease-associated variants and their impact on G5G8 structure–function.

## 1. Discovery to Therapy

In 1974, Bhattacharyya and Conner described a new lipid storage disorder in two sisters who presented with tendon and tuberous xanthomas and elevated plasma levels of phytosterols, sitosterol, campesterol, and stigmasterol [1]. Absorption of radiolabeled β-sitosterol was reported to be thirty-five times greater than that of normal subjects. They named their new lipid disorder β-sitosterolemia (hereon referred to as sitosterolemia), but it would be another 26 years before the discovery of *ABCG5 ABCG8* as the causative gene defect. Subsequent case reports established recessive genetics of the disease and greatly expanded its potential clinical presentation, which may include elevated low density lipoprotein (LDL) cholesterol, premature coronary artery disease and death, hemolytic anemia, macrothrombocytopenia, splenomegaly, adrenal dysfunction, elevated liver function tests, and cirrhosis [2,3,4,5,6,7,8,9,10,11,12,13]. Clinical studies in individuals with sitosterolemia revealed reductions in cholesterol synthesis, biliary cholesterol secretion, plasma clearance, and fecal elimination of neutral sterols [8,9,14,15]. Despite the absorptive phenotype and metabolism of phytosterols to bile acids, the clinical management of these patients with low sterol diets and bile acid binding resins resulted in modest and inconsistent reductions in plasma phytosterols [5,16].

Following the elimination of key genes in the esterification, absorbance, biosynthesis, and regulation of cholesterol metabolism, the sitosterolemia locus was mapped to a 0.5 centimorgan region on chromosome 2p21 [17,18]. The breakthrough would come two years later when investigators were studying agonists for liver X receptors (LXR-α NR1H3, LXR-β NR1H2), sensors of excess cholesterol that promote cholesterol mobilization from macrophages, metabolism to primary bile acids, and the excretion of neutral and acidic sterols [19,20]. A microarray screen of liver and intestinal transcripts from mice treated with an LXR agonist (T0901317) revealed a modest induction (2.5-fold) of the mouse brown gene, so named as it is the ortholog of the ABC transporter that determines brown eye color in *Drosophila* [21,22]. The human ortholog to mouse brown mapped to 2p21, *STSL* (OMIM: *STSL1*: 210250/*STSL2*:618666). At virtually the same time, independent groups identified individuals in multiple kindreds harboring nonsense mutations in either *ABCG5* or *ABCG8* with sitosterolemia, but not their unaffected family members [22,23]. *STSL1* and *STSL2* encode a pair of ATP binding cassette (ABC) half transporters in the G-subfamily. They reside on opposite strands of the DNA with initiation codons separated by a mere 374 base pairs. In these early reports, transcripts were restricted to the liver and intestine, the abundance of which increased in response to dietary cholesterol, suggesting the function of the transporters was to oppose intestinal absorption and promote biliary secretion of neutral sterols.

**Figure 1:**
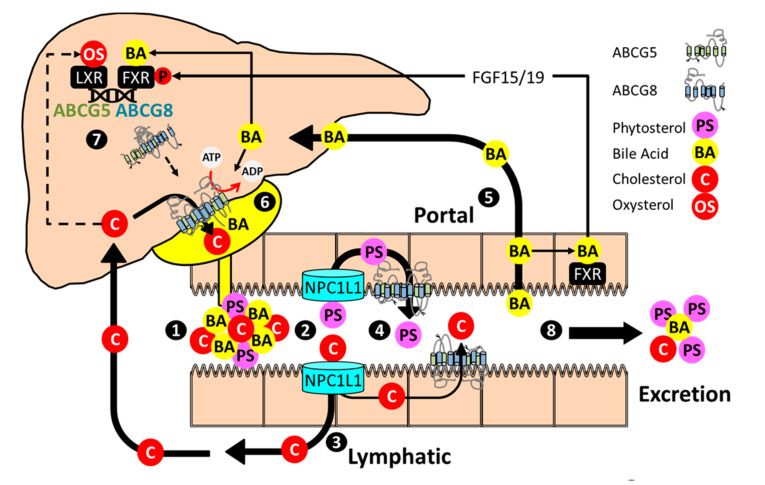
Enterohepatic sterol flux and regulation of *ABCG5 ABCG8*. (1) Bile acid micelles facilitate the solubilization of dietary and endogenous sterols in the proximal small intestine. Phospholipids not depicted. (2) NPC1L1 facilitates uptake of cholesterol and phytosterols into intestinal enterocytes (3) Cholesterol is incorporated into chylomicrons, delivered to the plasma compartment through the lymphatic system, and cleared by the liver. ABCG5 ABCG8 also promotes cholesterol secretion into the intestinal lumen. (4) Phytosterols are poorly absorbed and largely returned to the intestinal lumen by ABCG5 ABCG8. (5) Bile acids are reabsorbed in the distal small intestine, stimulate FXR-dependent expression of FGF15/19, and are returned to the liver through the portal system. (6) In the liver, bile acids stimulate ABCG5 ABCG8 catalytic activity and promote the formation of bile acid micelles that serve as acceptors for ABCG5 ABCG8 mediated biliary cholesterol secretion. (7) Cholesterol metabolites (oxysterols), through LXR, and bile acids, through FXR and in cooperation with FGF15/19, activate ABCG5 ABCG8. The half transporters heterodimerize, traffic to the canalicular surface, and promote biliary phytosterol and cholesterol secretion. (8) Excess cholesterol, phytosterols and bile acids that are not absorbed/reabsorbed are eliminated from the body.

The discovery of ezetimibe as the inhibitor of Neiman–Pick C-1-Like 1 (NPC1L1), and cholesterol absorption was a breakthrough in the clinical management of sitosterolemia [24,25]. Ezetimibe (10 mg/day) was tested as a cholesterol-lowering agent in healthy subjects with moderate hypercholesterolemia. A detailed analysis of plasma sterols revealed that in addition to cholesterol, plasma phytosterols were also reduced, indicating that phytosterols and cholesterol shared a common, ezetimibe-sensitive pathway for absorption, thus suggesting the drug might be effective in the treatment of sitosterolemia (Figure 1 (1)(2)). Ezetimibe was subsequently shown to reduce plasma phytosterols in sitosterolemic subjects by >20% after eight weeks [26]. In a two-year follow-up study, plasma sitosterol and campesterol levels were reduced by 44% and 51%, respectively [27]. It should be noted that phytosterol levels in these subjects remained well above normal. However, the reductions observed with ezetimibe as a single agent or as an adjunctive therapy resolves many of the clinical manifestations of sitosterolemia (reviewed in [28]).

## 2. Animal Models of Sitosterolemia

Three independent mouse models of sitosterolemia have been developed which lack functional *Abcg5*, *Abcg8*, or both half transporters [29,30,31]. In each model, the G5G8 heterodimer is absent. These models largely phenocopy one another and share many phenotypes with sitosterolemia in humans. These include elevated plasma and tissue levels of phytosterols, reduced biliary cholesterol, and repression of the cholesterol biosynthetic pathway. Since the discovery of *ABCG5 ABCG8*, phenotypes in spontaneous rodent models have been attributed to defects in *Abcg5 Abcg8* or the accumulation of phytosterols. The Spontaneous hypertensive rat (SHR) harbors a glycine 583 cysteine mutation in *Abcg5*, which segregates with elevated phytosterol levels in plasma, but not hypertension [32]. Similarly, a premature stop codon in *Abcg5* is present in the thrombocytopenia and cardiomyopathy (trac) mouse [33]. Plasma phytosterols and platelet counts were rescued by crossing this strain with mice harboring a human *ABCG5 ABCG8* transgene. Mice with a targeted disruption in either *Abcg5* or *Abcg8* also display platelet dysfunction, effects that are reversed with ezetimibe or low phytosterol-containing diets, respectively [34,35]. Other phenotypes in mice lacking functional G5G8 are reversed by ezetimibe treatment, genetic inactivation of its pharmacological target, Neiman–Pick C1-Like-1 (NPC1L1), or being fed a diet that lacks phytosterols [36,37,38]. Conversely, feeding diets enriched in phytosterols exacerbate phenotypes and results in sudden death [36,39].

Collectively, the available data indicates that the presentation of sitosterolemia in both humans and rodent models is a function of the type and abundance of xenosterols present in the diet that ultimately accumulates in plasma and tissues. This complicates interpretations of *ABCG5 ABCG8* physiology with respect to cholesterol metabolism, as phytosterols are known to produce a myriad of biological effects, including disruptions of sterol sensing by sterol receptor element binding protein 2 (SREBP-2), LXR, and the bile acid receptor (farnesoid X receptor, FXR NR1H4) [40,41,42,43].

While sitosterolemia may present with normal or only modestly elevated plasma cholesterol, cholesterol levels are generally lower in mice lacking functional G5G8 than their wild-type counterparts. The precise mechanism accounting for this difference has not been investigated but may be due to the paucity of ApoB containing lipoproteins in plasma in mice compared to humans. An alternative explanation is a species difference in the role of G5G8 in cholesterol absorption. Given its abundant expression in the intestinal epithelium, its role in biliary cholesterol secretion, and the common pathway for cholesterol and phytosterol absorption (NPC1L1), it stands to reason that G5G8 would oppose cholesterol absorption. However, studies in both humans and mice have produced inconsistent results. In both humans and mice, phytosterol absorption is clearly elevated [30,31,44,45]. Sitosterolemics appear on the higher end of the range for cholesterol absorption in humans, a trait that maps to the *ABCG5 ABCG8* locus [18,23]. However, the magnitude of the increase is relatively modest compared to the increase in the absorption of phytosterols. Mice lacking G5G8 do not show substantial increases in cholesterol absorption as assessed by the dual isotope method [29,31]. When monitoring the appearance of radiolabeled sterol in lymph, the absence of G5G8 either reduced or increased cholesterol absorption across the intestinal epithelium [46,47,48]. Expression of a human transgene under the control of its own promoter reduced fractional cholesterol absorption by 50% [49]. Consequently, the role of G5G8 in cholesterol absorption remains unclear, and like other phenotypes, may depend on dietary phytosterols.

Deletion of *Abcg5 Abcg8* reduces biliary cholesterol secretion by 70–90%. Agents that stimulate biliary cholesterol secretion are generally G5G8-dependent, including LXR and FXR agonists, thyroid hormone, and choleretic agents (diosgenin, tauroursodeoxycholate) [29,48,50,51,52,53,54]. Heterologous expression of Niemann–Pick C2 (NPC2) increases biliary secretion of the protein and promotes G5G8-dependent biliary cholesterol secretion, suggesting a role for this sterol-trafficking protein as a mediator of G5G8 activity or perhaps as a source of, or acceptor for G5G8 substrates [55]. G5G8-independent biliary cholesterol secretion is observed under some experimental conditions, including depletion and overexpression of class B, type-1 scavenger receptor (SR-BI), infusion or feeding high levels of cholate, *Atp8b1* deficiency, and in lactating rats [29,56,57,58,59,60]. Some fraction of residual biliary cholesterol secretion in the absence of G5G8 is likely mediated by detergent extraction. The extent to which other enzymes contribute to a G5G8-independent pathway, and if such a pathway might be targeted to increase biliary cholesterol secretion remains unknown.

## 3. Heterologous Expression of G5G8

The first *ABCG5 ABCG8* transgenic strain contained an estimated 14 copies of the human gene, increased biliary cholesterol concentrations five to six-fold, and reduced susceptibility to experimental hypercholesterolemia and atherosclerosis [49,61]. A liver-specific transgenic failed to protect mice from atherosclerosis unless combined with the cholesterol absorption inhibitor ezetimibe [62,63]. The protective effects of G5G8 are presumably due to its role as the mediator of the final step of reverse cholesterol transport (RCT). Pharmacological stimuli of RCT, including ezetimibe and LXR and FXR agonists, require G5G8 to promote fecal neutral sterol loss and macrophage to feces RCT [50,64,65]. However, acute adenoviral-mediated overexpression of hepatic G5G8 fails to stimulate macrophage to feces RCT [56]. Further, adenoviral expression of G5G8 paradoxically increases plasma cholesterol, an effect blocked by ezetimibe [66]. This indicates that a substantial amount of biliary cholesterol is reabsorbed in the small intestine and illustrates the cooperative nature of hepatic and intestinal G5G8 in order to oppose hypercholesterolemia and promote RCT. This, however, is not the case for preventing phytosterolemia as tissue-selective deletion of intestinal or hepatic G5G8 results in only modest elevations in plasma phytosterols [67].

## 4. Beyond Phytosterols

Given the roles of G5G8 in opposing dietary sterol accumulation and biliary cholesterol secretion, it is unsurprising that both rare and common variants have been associated with plasma cholesterol, non-cholesterol sterols, low-density lipoprotein cholesterol (LDL-C), and atherosclerotic and gallbladder disease across a large number of studies and populations ([68,69,70,71,72,73,74,75,76,77,78,79,80], incomplete list). Other associations are intriguing and include insulin resistance (G8:D19H) and type 2 diabetes (G5:G604E, G8:Y54C) and its renal complications (G8:T400K) [81,82,83]. Mouse models of metabolic syndrome which lack leptin or its receptor have diminished hepatic G5G8 and reduced biliary cholesterol secretion [66,84]. Rescue of G5G8 in these models with chemical chaperones, adenoviral expression of the molecular chaperone GRP78/binding immunoglobulin protein (BiP), or adenoviral expression of G5G8 itself accelerates biliary cholesterol secretion, restores glycemic control, and reduces plasma triglycerides [66,84,85]. Conversely, mice lacking G5G8 are more susceptible to diet-induced obesity, insulin resistance, and hepatic steatosis when maintained on phytosterol-free diets [86]. Collectively, these studies suggest an unappreciated relationship between biliary cholesterol secretion, triglyceride metabolism, and insulin signaling. There also appears to be a role for intestinal G5G8 in the absorption of triglycerides and chylomicron assembly, which may influence metabolic phenotypes, but the underlying mechanism for this phenotype remains unclear [46,47].

Independent of phytosterol accumulation, genome-wide association studies (GWAS) and animal model data support an anti-atherosclerotic role for G5G8. This is presumably due to the combined effects of limiting dietary cholesterol accumulation and promoting RCT. Classically, the final steps of RCT are hepatobiliary secretion of neutral and acidic sterols. However, disruptions in biliary cholesterol secretion do not result in concomitant reductions in cholesterol excretion, indicating the presence of a compensatory, non-biliary pathway which has been labeled transintestinal cholesterol elimination (TICE) [87]. Tissue-specific deletion of G5G8 in the intestine reduced excretion of radiolabeled sterol from the plasma compartment to feces, indicating a role for intestinal G5G8 in cholesterol excretion [67]. The mediators of TICE and the relative contribution of G5G8 to intestinal cholesterol excretion under a variety of pharmacological conditions remain to be fully elucidated. These studies and the potential for therapeutic development have recently been reviewed and are beyond the scope of this discussion [88,89].

## 5. Transcriptional Regulation of G5G8

*ABCG5 ABCG8* is effectively a single gene with a common promoter that regulates expression of both transcripts encoding each half of the transporter. To the best of our knowledge, there are no reports of differential regulation of *Abcg5* and *Abcg8* transcripts. Transcriptional regulation of *Abcg5 Abcg8* by a small molecule LXR agonist (T0901317) precipitated its discovery as the defective gene in sitosterolemia [22]. Expression of both mRNA and protein increases in liver and intestine in response to small molecule LXR agonists and dietary cholesterol, which promotes the accumulation of endogenous LXR agonists, oxysterols (Figure 1 (7)) [22,29,30,31,49,90]. Liver receptor homolog 1 (LRH1), GATA binding factor 4 (GATA-4), and hepatocyte nuclear factor 4α (HNF4α) binding sites map to the 374 base pair intergenic promoter that separates the initiation codons for each protein, the latter of which synergize with LXREs located in distal regions of the gene, to activate the promoter and increase expression of both transcripts [91,92]

Hepatic expression of *Abcg5 Abcg8* is also induced by FXR agonists and bile acids, and where examined, in an FXR-dependent fashion (Figure 1 (7)) [54,93,94,95]. However, regulation by bile acid FXR agonists is far more complicated. FXR-mediated activation of *Abcg5 Abcg8* requires fibroblast growth factor 15/19 (FGF15/19), which is itself an FXR target gene that is secreted from the ileum in response to bile acids and promotes Src-mediated phosphorylation of hepatic FXR, and FXR binding to the *Abcg5 Abcg8* promoter [96]. *FNDC5*/Irisin is an FXR target gene that increases *Abcg5 Abcg8* mRNA in both the livers and intestines of transgenic mice, but the extent to which the endogenous gene plays a role in *Abcg5 Abcg8* regulation and sterol homeostasis mice or humans is not known [97]. Whereas cholesterol and its metabolites tend to increase expression of *Abcg5 Abcg8* mRNA, agonists for the constitutive androstane receptor (CAR) repress expression of the transporter under conditions of elevated exogenous or endogenously-derived FXR agonists [98,99].

Transcriptional regulation of *Abcg5 Abcg8* by LXR and FXR fits well with its central role in opposing the accumulation of excess cholesterol. Expansion of whole body neutral and/or acid sterol pools increases expression [31,54,84,93,94,100,101]. Conversely, blocking absorption of cholesterol or bile acids reduces expression in either liver or intestine [37,101,102,103]. Less clear is the physiological benefit, if any, for alterations in *Abcg5 Abcg8* expression by regulators of metabolism. Thyroid hormone increases *Abcg5 Abcg8* mRNA, biliary cholesterol secretion, and fecal sterol excretion in both intact and hypophysectomized rats [104]. Hepatic *Abcg5 Abcg8* mRNA and protein are upregulated in the absence of insulin signaling in mice, an effect attributed to disinhibition of Forkhead box protein O1 (FOXO-1) [105,106]. The opposite was observed in a type 1 diabetic model in rats [107]. The insulin-sensitizing drug, metformin, increased *Abcg5 Abcg8* mRNA and protein, an effect attributed to reduced period 2 occupancy of the *ABCG5 ABCG8* promoter and disinhibition of gene expression [108]. Indeed, *Abcg5 Abcg8* mRNA exhibits a robust circadian rhythm at the transcriptional level (not observed for protein level, unpublished observation) and hepatic *Abcg5 Abcg8* mRNA and biliary cholesterol are reduced in *Bmal1*-deficient mice [108,109]

Alterations in *Abcg5 Abcg8* expression have been reported by a variety of nutritional cues, including upregulation in response elevated n-3 polyunsaturated fatty acids [110,111,112]. While upregulation of *ABCG5 ABCG8* is generally observed in high fat diets containing cholesterol and cholesterol-free, high fat diets, a single oral gavage of triacylglycerols robustly repressed intestinal *Abcg5 Abcg8* mRNA in mice [113]. In an independent study, suppression of *Abcg5 Abcg8* mRNA following high fat, high sucrose feeding was not observed, but the diet used in this study contained both added cholesterol and cholate, and thus hepatic cholesterol was increased five-fold compared to mice fed the control diet [114]. Diets containing high levels of sucrose robustly repressed expression of hepatic, but not intestinal *Abcg5 Abcg8* in rats [115]. Collectively, the data suggests that dietary repression of *ABCG5 ABCG8* may reflect reductions in the abundance of LXR and FXR agonists rather than active repression by sucrose or triglyceride. Alternatively, differences across studies may be associated with species and strain differences, both of which have been reported for various ligands [116,117]. Diets supplemented with soy protein increased hepatic *Abcg5 Abcg8* mRNA in rats [118]. The mechanisms for such an effect is not known but may include modulation of the intestinal microbiota. Germ free mice exhibited elevations in both intestinal and hepatic *Abcg5 Abcg8* mRNA relative to specific pathogen free mice in the absence and presence of ezetimibe [119]. Depletion of dietary iron upregulated hepatic *Abcg5 Abcg8* mRNA and promotes increased biliary cholesterol secretion [120]. Dietary calcium supplementation was shown to increased intestinal *Abcg5 Abcg8* mRNA and fecal neutral sterol excretion in a hamster model of menopause [121].

Female biological sex has long been associated with increased biliary cholesterol. Female mice had modest, but significant increases in biliary cholesterol and *ABCG5 ABCG8* mRNA in the human transgenic strain [49]. Ovariectomy was subsequently shown to reduce, and estrogen replacement to increase *Abcg5 Abcg8* mRNA across the intestine in independent strains of mice [116,122]. Diosgenin is a choleretic compound with estrogenic properties that increases biliary cholesterol secretion. Its ability to increase biliary cholesterol is largely G5G8-dependent, but reports on its impact on *Abcg5 Abcg8* expression are conflicting, showing no change in mice but an increase in both liver and intestine in rats [54,123]

## 6. Post-Transcriptional Regulation

Less is known about the post-transcriptional regulation of the G5G8. The half transporters are retained within the endoplasmic reticulum (ER) unless co-expressed [124,125]. Formation of the complex appears to be relatively inefficient in cultured cells, is dependent upon the presence of N-linked glycans that reside in the third extracellular loop of each protein, and can be enhanced by the expression of the lectin chaperones, Calnexin and Calreticulin [124,125,126,127]. Using chimeric approaches, the ER-retention motif was localized to the N-terminal, cytosolic domain, but has yet to be defined [128]. Failure to form complexes within the ER results in rapid degradation of each half transporter [127,129]. At the cell surface, the mature G5G8 complex resides within apical membranes of both hepatocytes and enterocytes [124]. There is also evidence of an intracellular, recruitable pool of G5G8 that translocates to the canalicular surface in response to cAMP and in response to diets containing cholate and cholesterol [114,130]. However, the stimuli and signaling pathways involved in intracellular trafficking of G5G8 have yet to be elucidated.

A number of approaches have been utilized to investigate the activity of G5G8. Heterologous expression in HEK293 and dog gall bladder epithelial cells demonstrated G5G8-dependent cholesterol efflux to bile acid micelles, but not HDL or apolipoprotein A1 [131,132]. Native mouse and recombinant human and mouse G5G8 have been purified to varying degrees from liver, rat hepatocytes, Sf9 insect cells, and *Pichia pastoris* [133,134,135,136]. These studies demonstrated ATP- and magnesium-dependent, vanadate-sensitive ATPase, and sterol transport activity. Various bile acids stimulate ATP hydrolysis. Among the species tested, G5G8 activity was most sensitive to cholate [133]. Perhaps surprisingly, neither cholesterol nor phytosterols stimulated ATPase or sterol transport activity in preparations from Sf9 cells [135,136]. Using inside-out vesicles in this same system, Wang et al. showed that other nucleotides could support sterol transfer, albeit less efficiently [135]

The nucleotide binding sites of G5G8 were proposed, and later confirmed by crystallography, to be comprised of Walker A and B domains of one partner and the signature motif of the other [137,138]. The Walker A and B domains of G8 juxtaposed to the signature motif of G5 were designated nucleotide binding site (NBS) 1. While both NBSs bind 8-Azido ATP, mutations in highly conserved residues within the Walker A and B domain of G5 (NBS2), but not G8 (NBS1), abolished ATP binding and hydrolysis. These findings were confirmed for G5G8-mediated biliary cholesterol secretion by expressing the mutants in G5G8-defecient mice. Domain swapping experiments between G5 and G8 confirmed that ATP hydrolysis in NBS2 is indispensable for activity [139]. G5G8 was crystallized as a heterodimer in lipid bilayers (bicelles) in the presence of cholesterol in the nucleotide free state to a resolution of four angstroms [138]. G5G8 was designated as a Type II Exporter. Key molecular interactions inferred from this structure were validated as essential for cholesterol transport in vivo by expressing recombinant mutants in G5G8-deficient mice. Naturally occurring missense variants and mutants can provide mechanistic insight to protein structure–function. The potential impact of mutations and polymorphisms on G5G8 structure function inferred from the available crystal structure were recently reviewed [140]. However, formal investigations into the impact of missense variants of any type on G5G8 trafficking, stability, and activity have been limited to only a few.

## 7. Sitosterolemia and/or Familial Hypercholesterolemia

Challenges in the proper diagnosis of sitosterolemia include heterogeneity of the clinical presentation of the disease, the lack of genotype–phenotype correlations, and the inability of clinical laboratory assays to distinguish phytosterols from cholesterol [28,141,142]. Studies among hypercholesterolemic subjects suggest sitosterolemia is significantly underdiagnosed [143,144]. Genome and exome sequence analysis of large populations indicates that carriers of loss of function mutations are far more common than previously thought and are at an elevated risk of coronary artery disease [72,80]. Exome sequence analysis of over 60,000 individuals across multiple populations reveals 57 and 58 predicted loss of function alleles for *ABCG5* and *ABCG8*, respectively (https://gnomad.broadinstitute.org/ (accessed on 3 January 2021) v2.1.1) [145]. This analysis did not include missense variants, 37 of which have been described for sitosterolemia [141]. At the time of the preparation of this review, 619 and 1307 missense variants have been catalogued in dbSNP for *ABCG5* and *ABCG8*, respectively. Most are predicted to be benign, but virtually none of those likely to be pathogenic or of uncertain significance have been experimentally or clinically validated. The number of *ABCG5 ABCG8* variants will undoubtedly grow as additional genomes and exomes are sequenced, as will the need for better tools to predict which variants are pathogenic.

An analysis of selected missense mutants of *ABCG5* and *ABCG8* suggests that the majority of dysfunctional alleles are due to the inability of *ABCG5 ABCG8* to heterodimerize and traffic beyond the endoplasmic reticulum [127]. The development of correctors and potentiators of the cystic fibrosis transmembrane conductance regulator (CFTR, *ABCC7*, OMIM: 602421) and the rescue of mutants of *ABCB4*, defective in progressive familial intrahepatic cholestasis type 3 (PFIC3, OMIM: 602347), suggest these or perhaps other small molecule chaperones may facilitate maturation and rescue function of G5G8 [146,147]. However, no systematic approach to classify the types of mutations that cause sitosterolemia has been made. We propose a draft of such a classification system for sitosterolemia (Table 1). We based our initial draft on the system established for PFIC3 due to the fact that both are biliary lipid transporters [148]. Our system may need future revision as additional mutations are identified and the impact of these variants/mutants on G5G8 formation, trafficking, and activity are determined. Nonetheless, this classification system provides a framework for characterizing *ABCG5 ABCG8* mutants that cause sitosterolemia and a basis for the systematic investigation of compounds that may potentially rescue G5G8 function.

## 8. Conclusions and Future Directions

The last twenty years have revealed a great deal about the role of *ABCG5 ABCG8* in cholesterol metabolism and in the defense against the accumulation of dietary xenosterols. The master regulators of neutral and acidic sterol metabolism modulate G5G8 abundance and activity as a component of the integrated machinery that maintains sterol homeostasis. Much, however, remains unknown about the hormonal and intracellular signals that promote G5G8 translocation to the biliary surface and G5G8-mediated cholesterol secretion. Beyond G5G8, the hepatocyte orchestrates the clearance of excess cellular cholesterol by metabolism to bile acid or incorporation into very low density and high-density lipoproteins, either on the surface of the particles or in the hydrophobic core following esterification. What regulates and under what conditions does the intracellular flux of cholesterol favor G5G8-dependent biliary secretion? Investigations of intestinal G5G8 regulation and activity have been more limited than in the liver. In what ways does the regulation of G5G8 in the enterocyte differ, if any, from the hepatocyte? Targeting G5G8 to promote TICE is conceptually attractive to promote RCT, but studies have yet to reveal a route to a potential therapeutic and the molecular mechanisms that mediate TICE remain elusive.

Sequencing of large numbers of genomes and exomes reveals that disease-causing mutants in *ABCG5 ABCG8* are significantly more common that previously appreciated. The combination of the required instrumentation, expertise, and cost for routine clinical laboratory analysis of plasma xenosterols is presently impractical. Conversely, genetic screening has become substantially less costly and increasingly common across healthcare systems. Genetic testing may offer a more practical means to identify and diagnose sitosterolemics. Such an approach requires the cataloguing and inclusion of *ABCG5 ABCG8* mutants among the various genetic testing platforms in use. A major limitation for such an approach is the lack of validation of suspected and likely pathogenic variants that may disrupt G5G8 function. Frameshift mutations in exon 13 of both *ABCG5* and *ABCG8* cause sitosterolemia, indicating little tolerance for truncation of the protein. Thus, deletions or frameshifts are expected to be Class I mutants. A significant number of splice donor and acceptor variants have also been identified and are likely pathogenic, but have yet to be formally analyzed. Of the 37 known missense variants, only 13 have been analyzed for maturation. Although most failed to form mature complexes and have been designated as Class II, three retained at least some degree of G5G8 maturation (G5:E146Q, G8:R543S, G8:G574R). Future investigations of the structural and functional impact of these and other missense mutants and variants will advance our understanding of sterol transport, the molecular dynamics of the transporter, and potential therapeutics for sitosterolemia as well as gall bladder and cardiovascular risk reduction.

## Figures and Tables

**Table 1:** Classification system for experimentally verified sitosterolemia mutations.

Typical	No Mutation	ABCG5	ABCG8
Class I	Nonsense, Frameshift, Deletions	57 known or predicted	58 known or predicted
Class II	Maturation	R389H, R419P, R419H, N437K	R189H, P231T, R263Q, L501P, G574E, L596R
Class III	Activity		
Class IV	Stability		
Class V	No Detectible Defect		
Unclassified	Inconclusive Results	E146Q	R543S, G574R

https://gnomad.broadinstitute.org/ (accessed on 3 January 2021) and Reference [127].
